# Supplementary material for: Influence of Atmospheric Pollutants on Allergic Sensitization to Cupressaceae, Olea, and Platanus Pollen in the Community of Madrid (2017–2021)
Source: Life (Basel). 2025 Nov 19;15(11):1774. doi: 10.3390/life15111774 (PMC12653589; doi:10.3390/life15111774)
Supplement: Supplementary file 1 [file life-15-01774-s001.zip › life-3950248-Supplementary.pdf]

life

MDPI

Article

Influence of Atmospheric Pollutants on Allergic Sensitization to Cupressaceae, *Olea*, and *Platanus* Pollen in the Community of Madrid (2017–2021)

Javier Chico-Fernández [1,*], Angélica Feliu Vila [2], Beatriz Rodríguez-Jiménez [3], Teresa Valbuena Garrido [4] and Esperanza Ayuga-Téllez [5,*]

[1] Programa de Doctorado Ingeniería y Gestión del Medio Natural, ETSI de Montes, Forestal y del Medio Natural, Universidad Politécnica de Madrid, 28040 Madrid, Spain
[2] Servicio de Alergología, Hospital Universitario del Tajo, 28300 Madrid, Spain; angelica.feliuvila@salud.madrid.org
[3] Servicio de Alergología, Hospital Universitario de Getafe, 28905 Madrid, Spain; brodriguezj@salud.madrid.org
[4] Sección Alergología, Hospital Universitario Infanta Sofía, FIIB HUIS-HUHEN, 28702 Madrid, Spain; mteresa.valbuena@salud.madrid.org
[5] Buildings, Infrastructures and Projects for Rural and Environmental Engineering (BIPREE), Universidad Politécnica de Madrid, 28040 Madrid, Spain
* Correspondence: javier.chico@alumnos.upm.es (J.C.-F.); esperanza.ayuga@upm.es (E.A.-T.)

Academic Editor: Daniele Focosi

Received: 10 October 2025
Revised: 15 November 2025
Accepted: 16 November 2025
Published: 19 November 2025

Citation: Chico-Fernández, J.; Feliu Vila, A.; Rodríguez-Jiménez, B.; Valbuena Garrido, T.; Ayuga-Téllez, E. Influence of Atmospheric Pollutants on Allergic Sensitization to Cupressaceae, *Olea*, and *Platanus* Pollen in the Community of Madrid (2017–2021). *Life* **2025**, *15*, 1774. https://doi.org/10.3390/life15111774

Copyright: © 2025 by the authors. Licensee MDPI, Basel, Switzerland. This article is an open access article distributed under the terms and conditions of the Creative Commons Attribution (CC BY) license (https://creativecommons.org/licenses/by/4.0/).

Abstract: Tree pollen is the most abundant in the Community of Madrid (CAM), and specifically, pollen types from *Olea*, Cupressaceae, and *Platanus* are the most allergenic, after Gramineae, in this Spanish region. Air pollutants are one of the most significant stress factors for wind-pollinated vegetation, especially in urban areas, and can cause alterations in the immune system and the consequent triggering of type I hypersensitivity reactions mediated by immunoglobulin E (IgE). This study analyzes the allergic sensitization caused by the interrelation of O_3, NO_2, and PM_{10} pollutants with the tree pollen types *Olea*, Cupressaceae, and *Platanus* in the period 2017-2021. To this end, general linear models were calculated using the Statgraphics Centurion 19 tool. The data collected came from the Air Quality Networks of the CAM and Madrid City Council, the CAM Palynological Network, and the Allergy Services of the reference hospitals in the five study areas. This research confirms a statistically significant correlation between allergic sensitivity to pollen types and their concentrations in the air, and those of atmospheric pollutants, in the different areas and years studied. These pollen and pollutant concentrations in the atmosphere of the CAM jointly influence the prevalence of allergic sensitisation, as is evident in all the models calculated.

Keywords: tree pollen; air pollution; pollen sensitization; pollinosis

S2.1. Allergy sensitisation data

In Spain, there has been an increase in the prevalence of pollen sensitisation, as well as allergens derived from animal epithelia, as confirmed by the latest editions of Alergológica [3], which leads to the conclusion that there is an increase in atopic diseases whose origin lies in these airborne allergens.

In fact, the percentage of positive results in pollen tests in patients with allergic asthma has risen from 43.8% in 2005 to 65.6% in 2015 (and in 1992 it was 39.8%). Although the different inclusion criteria, and the different geographical origins of the patients must be considered, as well as the limitations of the methodology used in the different editions

of this publication by the Spanish Society of Allergology and Clinical Immunology (SEAIC) [3].

In the case of Spanish asthma patients, the most sensitising pollen types are Gramineae (42.1%), followed by *Olea* (36.9%), Cupressaceae (26%), *Salsola Kali* (7.3%) and *Platanus* (7.0%) [3].

In the CAM, asthma patients are mainly sensitised to Gramineae pollen (61% sensitisation frequency), followed by *Olea* (34.7% according to Alergológica 2005, as this data is missing from the 2015 edition), Cupressaceae (26%) and *Platanus* (19%) sensitisation frequency [3].

Rhinoconjunctivitis, the most prevalent allergic disease, coexists with other allergic conditions in 68% of cases in Spain, but it is most commonly associated with asthma (the second most prevalent allergic condition) in 33.7% of patients [3]. Again, as with asthma, the aeroallergens that mainly cause allergic sensitisation are pollens (70.8% in 2015, compared to 51.9% in Alergológica 2005, a truly striking increase) [3].

The types of pollen that mainly cause allergic sensitisation in Spain are, in order, Gramineae (73.7%), *Olea* (52.1%), Cupressaceae (22.8%) and *Platanus* (14.2%). In the CAM, they are Gramineae (87.1%), *Olea* (58.9%), Cupressaceae (40.5%) and *Platanus* (23.9%) [3].

Therefore, tree pollen types are among the main causes of allergic sensitisation, both in the case of asthmatic patients of extrinsic origin and in those suffering from allergic rhinoconjunctivitis. Furthermore, the percentages of sensitisation to pollen types in general have increased from one edition of Alergológica to the next, as have those referring to the pollen types studied in the CAM [3].

S2.2. Tree data

Data has been collected from urban tree inventories in the five study areas, attempting to correspond to the period 2017-2021 or, failing that, as close as possible to those study years, to establish an adequate correlation with the data on allergic sensitivity and atmospheric concentration of both pollen and pollutants included in this research. In fact, as can be seen in Table S1, the tree data for the municipalities of Alcobendas and Getafe, as well as those for Madrid Barrio de Salamanca, correspond to the year 2025, as they have been updated very recently. The inventories for the latter two areas were provided through the respective websites of the municipalities of Getafe and Madrid. The tree data for Leganés was updated in December 2024. And the data for Aranjuez was updated in 2023 (Table S1).

Table S1. Details of the number of trees of the three tree taxa studied, as well as the total number of specimens of all species inhabiting the five geographical areas covered by this research. The percentage of each taxon with respect to the total is also specified, as well as the year in which each inventory was carried out. Population density is also specified, based on the surface area of each study area and the number of inhabitants according to the CAM Statistics Institute and the Madrid City Council's Sub-Directorate General for Statistics for the year 2024.

**Study areas**<br>(No. hbt/Terr. Ext. = dens. pob.)	**Cupressaceae**	***Olea***	***Platanus***	**Total**	**Year**
**Alcobendas**<br>(121,446 hbt/4,527 ha = 26.83 hbt/ha)	3,275<br>(8.28%)	364<br>(0.92%)	5,584<br>(14.12%)	39,540	2025
**Aranjuez**<br>(62,508 hbt/18,917 ha = 3.30 hbt/ha)	1,180<br>(4.62%)	679<br>(2.66%)	5,646<br>(22.10%)	25,546	2023
**Madrid: Barrio de Salamanca**<br>(149,778 hbt/539.24 ha = 277.76 hbt/ha)	1,030<br>(5.02%)	44<br>(0.21%)	2,439<br>(11.89%)	20,515	2025
**Getafe**<br>(191,560 hbt/7,869 ha = 24.34 hbt/ha)	4,692<br>(7.37%)	744<br>(1.17%)	4,544<br>(7.14%)	63,651	2025

**Leganés** (193,934 hbt/4,325 ha = 44.84 hbt/ha)	3,106 (4.81%)	651 (1.01%)	12,060 (18.68%)	64,569	2024

The trees included in these municipal statistics are located both on streets and in green areas within cities. Therefore, trees outside urban areas are not normally included. Nor are trees located in areas not managed by the respective local council. Such is the case of the Polvoranca forest park, in the municipality of Leganés, and the Bosquesur forest park, which, in addition to Leganés, extends linearly along the course of the Arroyo Culebro stream, through the municipalities of Fuenlabrada, Getafe and Pinto.

Both parks, together with those of Valdebernardo and La Cantueña, belong to the CAM's Network of Peri-urban Forest Parks and were created and are administered by the autonomous community, with the aim of acting as ecological corridors between each other and promoting biodiversity, while at the same time facilitating the enjoyment of nature in a geographically close location for citizens [24].

In the same situation, in terms of the criteria for inclusion in the urban tree study, is the town council of Aranjuez, whose inventory does not include the trees in the Jardín de la Isla, the Jardín del Príncipe and the Jardín del Rey, in the Royal Palace. In fact, these gardens are managed by Patrimonio Nacional [25].

Another noteworthy feature is that in the Madrid Barrio de Salamanca zone, all the trees in green areas and streets are included, as well as those in the historic Quinta de la Fuente del Berro park. In fact, for example, 154 of the 335 *Cupressus sempervirens* trees are located in this green area, as can be seen in the inventory provided by Madrid City Council.

On the other hand, specimens that have died and those of which only stumps remain have been removed from both the total number and the respective three tree taxa under study. Furthermore, in some municipal inventories, such as that of Getafe, young trees have been planted that have not yet been inventoried, so their species, dendrometric characteristics and, in some cases, their geographical location do not appear in the data files.

For this reason, they are included in the total count but not in the count by study taxa, which could increase the number of these specimens in these cases and alter the percentages shown in Table S1 upwards.

Within the Cupressaceae family, all existing trees have been counted from the five municipal inventories, including the most common species, i.e. those of the *Cupressus* genus, *Platycladus*, and *Cupressocyparis*, as well as those species that are rarer in urban areas, such as those of the *Juniperus*, *Sequoiadendron*, and *Tetraclinis* genera. In absolute terms, the municipality with the highest number of Cupressaceae trees, specifically 4,692, is Getafe, followed by Alcobendas, with 3,275 specimens, and Leganés, with 3,106.

Aranjuez and Madrid Barrio de Salamanca are the areas with the fewest trees of this family of gymnosperm conifers in their territory, with 1,180 and 1,030 trees, respectively (Table S1). However, in relative terms, in terms of the percentage of specimens in relation to the total recorded in the study areas, Alcobendas, with 8.28%, exceeds Getafe, with 7.37%, and Madrid Barrio de Salamanca (5.02%). Leganés and Aranjuez are the municipalities with the lowest percentage of Cupressaceae, with 4.81% and 4.62%, respectively, as can be seen in Figure S1 (and Table S1).

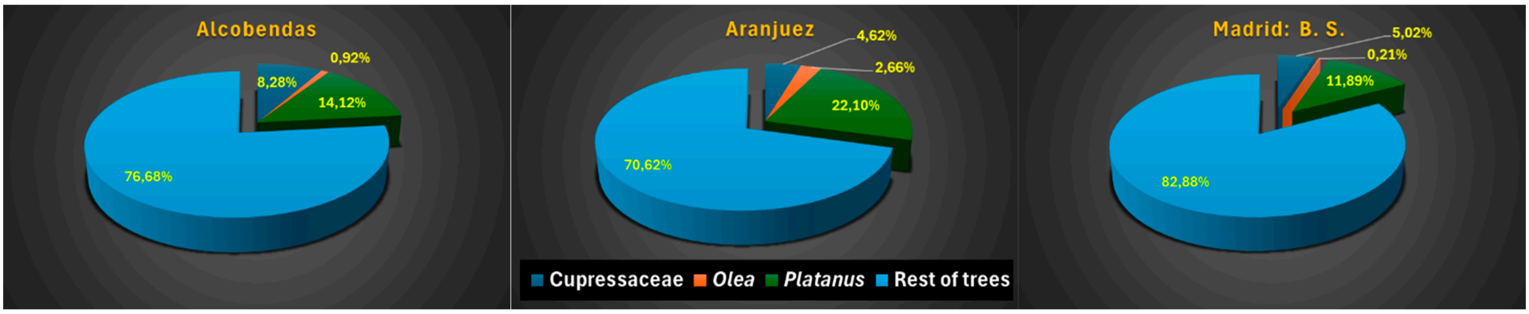

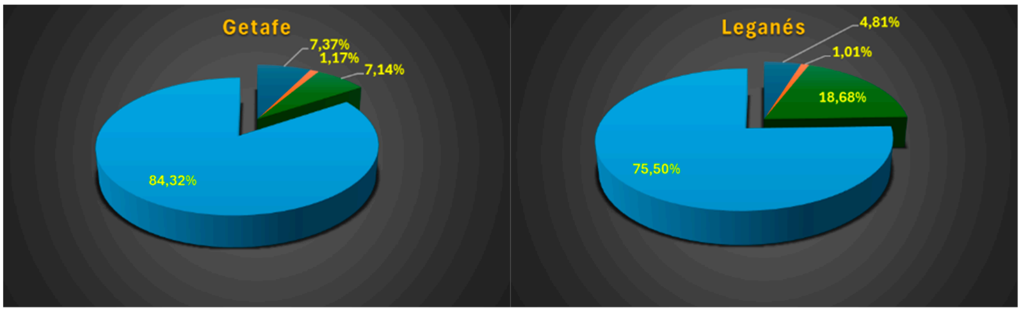

Figure S1. Relationship between the graphs showing the presence of the three tree taxa in relation to the total number of trees present in each of the five study areas. The proportion of other trees, which are not included in this study, is also specified.

In the case of *Olea*, the only species found in the five urban tree inventories is *Olea europaea*. The proportion of *Olea europaea* specimens in relation to the total number of trees inventoried in the five study areas is highest in Aranjuez, with 2.66%, followed by Getafe (1.17%), Leganés (1.01%) and Alcobendas (0.92%). Madrid Barrio de Salamanca has only 44 trees, representing just 0.21% of the total number of trees in that district of the city of Madrid (Figure S1, Table S1). However, in absolute terms, Getafe, with 744 specimens, exceeds Aranjuez in number of olive trees, with 679 trees, Leganés, with 651, and Alcobendas, with 364 specimens of this species (Table S1).

Finally, the presence of specimens of the genus *Platanus*, mainly consisting of the species *Platanus hispanica* (and with only a few specimens catalogued in the five study areas with the species *Platanus orientalis* and *Platanus occidentalis*), is greater in proportion to the total of 25,546 trees, in Aranjuez, with a significant 22.10%, followed by 18.68% in Leganés, 14.12% in Alcobendas, 11.89% in Madrid Barrio de Salamanca and 7.14% in Getafe (Figure S1, Table S1).

As can be seen in Table S1, in four of the five study areas, the highest absolute and therefore also relative presence (Figure S1) of the three tree taxa is found in *Platanus*, followed by Cupressaceae and *Olea*. The exception to this rule is Getafe, where, although there are 4,544 *Platanus* specimens in the area (representing 7.14% of the total of 63,651 trees inventoried in that municipality), this number is exceeded by that of Cupressaceae trees, 4,692, representing 7.37%, a percentage slightly higher than that mentioned for *Platanus*. However, it is true that the number of *Olea* specimens is lower than that of the other two tree taxa studied, a circumstance that occurs in all five study areas.

As can be observed from the data obtained from the CAM Statistics Institute on the land area of the different municipalities studied [26] and the total registered population as of 1 January 2024 [27], the municipality of Aranjuez, with 18,917 ha, is the largest in the CAM after the municipality of Madrid [26]. Its population density is only 3.30 inhabitants per hectare (hbt/ha), by far the lowest in relation to the other four areas under study (Table S1). At the opposite end of the scale is Madrid Barrio de Salamanca, with 277.76 hbt/ha (population density obtained from data provided by the Subdirectorate General of Statistics of the Madrid City Council [28]). Leganés has the second highest density, with 44.84 hbt/ha. The number of inhabitants per hectare in the towns of Alcobendas and Getafe is similar, with 26.83 and 24.34 hbt/ha, respectively (Table S1).

Furthermore, Table S1 shows that the municipality of Leganés has the highest number of trees, 64,569, in terms of its territory, despite having the second smallest area (4,325 ha), after the Madrid neighbourhood of Salamanca, which covers only 539.24 ha [26,28]. The municipality of Getafe is the second most wooded, with 63,651 trees planted by 2025, a number not far behind that of Leganés. However, Getafe has an area of 7,869 ha, significantly larger than that of Leganés [26].

Given that the trees under study are located on urban land, whether on streets or in public and private parks and gardens, the areas of this type of land have been consulted in the case of the municipalities of Leganés, Getafe, Alcobendas and Aranjuez, through the Directorate-General for Urban Planning of the Regional Ministry of the Environment, Land Planning and Sustainability of the CAM, and refer to the year 2017. This is the last year in which they were recorded from the planning files of the different municipalities of the CAM, processed for approval [29,30]. As for the Salamanca district of Madrid City Council, in December 2006 it had a consolidated urban land area of 529 ha, and the remaining 10 hectares of its total area consisted of land undergoing transformation, with '0' hectares of non-developable land [31].

The data for these areas are shown in Figure 1(in the main text), as well as the total number of trees in each study area, as specified in Table S1. Thus, Leganés and Getafe are the municipalities that lead the list in terms of total number of trees, far ahead of Alcobendas, Aranjuez and Madrid Barrio de Salamanca.

However, Leganés has an urban area of 1,011.73 ha, almost half that of Getafe (1,906.24 ha), which is why Leganés not only has a greater number of trees, but also, in proportion to its surface area, far exceeds Getafe. Alcobendas, however, has the largest urban area of the five areas studied, with 2,776.98 ha, more than 2.5 times the urban area of Leganés. Even so, with 39,540 trees, it is far from reaching the number of trees in that municipality (Figure 1, in the main text).

Madrid Barrio de Salamanca, has the smallest area in the comparison, with 539.24 ha, and the one with the fewest trees, 20,515. The municipality of Alcobendas is more than five times larger than this district of Madrid. However, in proportion, the Salamanca district of Madrid has a higher tree density than Alcobendas, as it has almost half the number of trees per unit area than this municipality. Furthermore, it should be noted that the population of the Madrid district is 149,778 inhabitants (with a population density of 277.76 hbt/ha), as can be seen in Table S1; these demographic figures are much higher than those of Alcobendas.

Finally, the municipality of Aranjuez has 552.61 ha, the second smallest area of urban land in the study areas. It also has the second smallest total number of trees, 25,546. However, proportionally, it has a slightly higher tree density than Madrid Barrio de Salamanca, which is much higher than that of Alcobendas and Getafe, and lower than that of Leganés (Figure 1, in the main text). It is true that, as mentioned above, Aranjuez has trees in its historic gardens, which are not included in the total of 25,546 trees, among which the Jardín del Príncipe alone covers an area of 150 ha [25].

References

3. SEAIC. Alergológica 2015; SEAIC: Madrid, Spain, 2017; ISBN 978-84-88014-41-2.
24. Parques forestales periurbanos. Available online: https://www.comunidad.madrid/servicios/urbanismo-medio-ambiente/parques-forestales-periurbanos (accesed on 16 July 2025)
25. Jardines de Aranjuez. Patrimonio Nacional. Available online: https://www.patrimonionacional.es/visita/jardines-de-aranjuez (accessed on 15 July 2025).
26. Territorio y Climatología. Extensión. Consejería de Medio Ambiente, Agricultura e Interior. Banco de datos municipal y zonal ALMUDENA. Instituto de Estadística Comunidad de Madrid. Available online: https://gestiona.comunidad.madrid/desvan/AccionDatosTemaMunicipal.icm?codTema=101001 (accesed on 11 July 2025)
27. Población total censada. Censo de Población Anual. INE. Banco de datos municipal y zonal ALMUDENA. Instituto de Estadística Comunidad de Madrid. Available online: https://gestiona.comunidad.madrid/desvan/AccionDatosTemaMunicipal.icm?codTema=1929381 (accesed on 11 July 2025)
28. Distritos en cifras (Información de Barrios). Subdirección General de Estadística Ayuntamiento de Madrid. Available online: https://www.madrid.es/portales/munimadrid/es/Distritos-en-cifras-Informacion-de-Barrios-/?vgnextfmt=default&vgnextoid=0e9bcc2419cdd410VgnVCM2000000c205a0aRCRD&vgnextchannel=27002d05cb71b310VgnVCM1000000b205a0aRCRD (accesed on 11 July 2025)

29. Usos del suelo y Planeamiento desde 2005. Suelo urbano. Dirección General de Urbanismo. Consejería de Medio Ambiente, Ordenación del Territorio y Sostenibilidad. Banco de datos municipal y zonal ALMUDENA. Instituto de Estadística Comunidad de Madrid. Available online: https://gestiona.comunidad.madrid/desvan/AccionDatosTemaMunicipal.icm?codTema=1905503&utm (accesed on 21 July 2025)
30. Planeamiento de suelo urbano. Dirección General de Urbanismo. Consejería de Medio Ambiente, Ordenación del Territorio y Sostenibilidad.Instituto de Estadística Comunidad de Madrid. Available online: https://gestiona.comunidad.madrid/desvan/GenerarPDF.icm?codTema=1905503 (accesed on 21 July 2025)
31. Estado de desarrollo de los ámbitos del plan general. Situación a diciembre 2006. Distrito Salamanca. Área de Gobierno de Urbanismo, Vivienda e Infraestructuras. Dirección General de Planificación y Evaluación Urbana. Available online: https://www.madrid.es/UnidadWeb/Contenidos/Publicaciones/TemaUrbanismo/SituacionSueloMunMadrid/Ficheros/SALAMANCA.pdf (accesed on 21 July 2025)

Disclaimer/Publisher's Note: The statements, opinions and data contained in all publications are solely those of the individual author(s) and contributor(s) and not of MDPI and/or the editor(s). MDPI and/or the editor(s) disclaim responsibility for any injury to people or property resulting from any ideas, methods, instructions or products referred to in the content.
